# Effect of Education on Impaired Hypoglycemia Awareness and Glycemic Variability in Children and Adolescents with Type 1 Diabetes Mellitus

**DOI:** 10.4274/jcrpe.galenos.2019.2019.0009

**Published:** 2019-05-28

**Authors:** Günay Demir, Samim Özen, Hafize Çetin, Şükran Darcan, Damla Gökşen

**Affiliations:** 1 Ege University Faculty of Medicine, Department of Pediatric Endocrinology and Diabetes, İzmir, Turkey

**Keywords:** Continuous glucose monitoring, education, impaired hypoglycemia awareness, glycemic variability, type 1 diabetes, children

## Abstract

**Objective:**

The aim of this study was to determine the prevalence of impaired hypoglycemia awareness (IHA) in children and adolescents with type 1 diabetes mellitus using a professional continuous glucose monitoring (CGM) system and to show the effect of structured education on glycemic variability (GV) in children and adolescents with IHA.

**Methods:**

Forty type 1 diabetic children and adolescents with a diabetes duration of at least five years were eligible for inclusion in this prospective, quantitative study. All subjects were asked about their history of being aware of the symptoms of hypoglycemia using a questionnaire. Professional CGM was conducted in all of the patients for six days. The frequency of IHA detected by comparison of CGM and logbook reports were analyzed. Patients with identified IHA underwent a structured training program. After three months, CGM was re-applied to patients with IHA.

**Results:**

The study was completed by 37 diabetic children and adolescents. After the initial CGM, nine patients (24.3%) were found to have had episodes of IHA. Area under the curve (AUC) for hypoglycemia and number of low excursions were; 1.81±0.95 and 8.33±3.60 for the IHA group at the beginning of the study. AUC for hypoglycemia was 0.43±0.47 after three months of structured education the IHA patients (p=0.01). Coefficient of variation which shows primary GV decreased significantly although unstable at the end of education in IHA patients (p=0.03).

**Conclusion:**

CGM is a valuable tool to diagnose IHA. IHA, GV and time in range can be improved by education-based intervention.

**What is already known on this topic?**

Impaired hypoglycemia awareness and glycemic variability are important problems causing acute and chronic complications in children and adolescents with type 1 diabetes.

**What this study adds?**

Professional continuous glucose measurement system is a valuable tool to diagnose impaired hypoglycemia awareness (IHA) in type 1 diabetic children and adolescents. IHA, glycemic variability and time in range can be improved by education-based intervention.

## Introduction

Hypoglycemia is the most common acute complication of type 1 diabetes with adverse effects on both the quality of life of patients and the management of their diabetes (1,2). Hypoglycemia is usually defined as a plasma glucose level <70 mg/dL (3.9 mmol/L) (3). The following classification of hypoglycemia, based on clinical evaluation, is worth considering (4). Level 1: a hypoglycemia alert glucose value of <70-54 mg/dL (3.9-3.0 mmol/L) with or without symptoms. Level 2: a glucose level of <54 mg/dL (<3.0 mmol/L) with our without symptoms. This glucose level should be considered clinically significant hypoglycemia requiring immediate attention. Level 3: severe hypoglycemia. This denotes cognitive impairment requiring external assistance for recovery but is not defined by a specific glucose value.

The main symptoms of hypoglycemia occur as a result of neuroglycopenic and autonomic activation (5). Neuroglycopenic symptoms occur as a result of hypoglycemic activation of the autonomic nervous system and these symptoms are often severe enough so that hypoglycemia will be noticed by the patient, thus providing protection from complications related to hypoglycemia (6). Nocturnal hypoglycemia is often asymptomatic and mild hypoglycemia during the day may also not be noticed by the patient. Therefore it is difficult to determine the true frequency of hypoglycemia. As efforts to achieve optimal glucose control increase in order to prevent the chronic complications of diabetes, the risk of hypoglycemia increases. Recurrent antecedent hypoglycemia induces sympathoadrenal responses and unawareness of hypoglycemia (6,7,8,9,10). This is known as impaired hypoglycemia awareness (IHA) and can be defined as the inability to perceive the onset of hypoglycemia.

Typically, autonomic symptoms are lost before neuroglycopenic symptoms, which then predominate (3). Type 1 diabetic patients with IHA and impaired counter-regulation are more likely to suffer from severe hypoglycemia, have longer diabetes duration and, interestingly, lower hemoglobin A1c (HbA1c). In addition IHA is a major limitation to achieving tight metabolic control of type 1 diabetes and reduced quality of life. The perception of adrenergic symptoms are reduced or disappear completely in these patients (6,7). It has been reported that careful glucose monitoring, individualized blood glucose targets and structured education programs are important in preventing and managing IHA (6,7,8,9,10). Real time continuous glucose monitoring (CGM) systems reduce IHA in children, adolescents and adults with type 1 diabetes (6,10).

The aim of this study was to determine the prevalence of IHA in children and adolescents with type 1 diabetes mellitus attending a single center by using a professional CGM system. A further aim was to examine the effect of structured education on glycemic variability (GV) in children and adolescents with IHA.

## Methods

Type 1 diabetic children and adolescents with a diabetes duration of at least five years were eligible for inclusion in this prospective, quantitative study. Patients were selected regardless of their metabolic control. The study was approved by the Ege University Medical Ethics Committee (approval number: 14-7/15). Written, informed consent was obtained from all participants and their parents.

All subjects were asked about their history of being aware of the symptoms of hypoglycemia prior to starting CGM with the following question: “Do you feel the symptoms of hypoglycemia”. Possible answers were: “yes”, “no” or “sometimes”. All subjects and their parents were invited to the outpatient clinic for a two hour training and evaluation session. CGM sensors used for all subjects were Medtronic iPro^®^2 professional CGM system (MiniMed Medtronic, Northridge, USA). Sensor placement was performed by one of the experienced Diabetes Educators. Calibration of the sensor was accomplished by following the protocol established and outlined in the MiniMed CGM manual.

During CGM, patients and parents were asked to measure a minimum of four finger-stick blood glucose levels per day and to record glucose values, meals, insulin doses, exercise periods and symptomatic hypoglycemia in a logbook. Patients used the same brand of glucometer during the monitoring period (Accu Chek performa Nano, Roche Diagnostics, Germany).

At the completion of the six-day CGM period, the system was returned and the data downloaded to determine glucose patterns together with data from the logbooks. Glucose data from each day were analyzed at two different time periods: day and night. Responses to hypoglycemia and exercise, the presence of unrecognized hypoglycemia and the number of high and low patterns seen with the CGM were evaluated from the information collected. Hypoglycemia was defined as a value below 70 mg/dL of glucose. Patients noted the events of symptomatic hypoglycemia occurring over the six days. These notes were compared with the data obtained from CGM.

Data on mean annual HbA1c values were obtained from medical records. HbA1c was measured by turbidimetric inhibition immunoassay (Roche Cobas c513 analyzer using the Tina quant^®^ HbA1c Gen. 3 assay, Germany) before the monitoring period and three months after modifications were made.

The frequency of IHA detected by compariosn of CGM and logbook reports were analyzed. Patients with IHA diagnosed by CGM underwent a structured training program (administration of insulin, hypoglycemia training, safe exercise management and ideal blood sugar levels) and the patients were seen weekly for three months. More frequent capillary blood glucose measurements were performed (4-6 times daily). After three months, CGM was re-applied to patients with IHA.

### Statistical Analysis

Data were evaluated using SPSS for Windows, version 16.0 statistical package program (IBM Inc., Chicago, IL., USA). Participants’ gender, nutrition, hypoglycemia insensitivity and hypoglycemia insensitivity according to their sex status, duration of grouped diabetes and hypoglycemia insensitivity to diabetes, and hypoglycemia symptoms were analyzed by chi-square test. HbA1c levels before and after the study, the number of blood glucose measurements at the beginning of the study and the t-test for independent groups were used for the analysis of the CGM at the beginning of the study. Mann-Whitney U test was used to analyze the baseline data of the participants. Wilcoxon sorting test was used for the analysis of the CGM data before and after the study. A p<0.05 was considered significant.

## Results

Forty patients were recruited for the study. Three patients withdrew because of poor sensor compliance. Thus the study was completed by 37 diabetic children and adolescents. Mean ± standard deviation age of the patients and mean diabetes duration were 13.80±2.42 and 7.67±1.66 years respectively. 41% were male, 59% were female. Mean HbA1c was 8.0±1.2% for the total group. Twenty five patients were on multiple daily insulin (MDI) therapy while the rest were on continuous subcutaneous insulin infusion (CSII) without sensor. No significant difference was found between CSII and MDI patients when comparing mean HbA1c at the start of therapy.

After the initial CGM, nine (six female) patients (24.3%) had episodes of IHA. Seven (77.7%) of the IHA patients were on MDI and two were on CSII. Six (66.6%) of the IHA patients had relatively shorter duration of diabetes (between five and eight years) while the remainder had a longer duration ranging from nine to eleven years. Seven (77.7%) of the IHA patients had completed puberty; one was Tanner stage 3 and the other Tanner stage 1. Mean HbA1c and glucose levels of the patients with and without IHA within the preceding year are given in Table 1.

Eight (21.6%) of the patients diagnosed as IHA with CGM filled out the questionnaire as ‘I always feel the symptoms’ and one (2.7%) of the patients who answered the questionnaire as ‘I sometimes feel the symptoms’ was diagnosed as IHA with CGM. There was no significant correlation between the true presence of IHA and the declared awareness of hypoglycemia, as given in the questionnaire responses.

IHA cases were hypoglycemic (blood glucose <70 mg/dL) for 11.44±5.12 hours while patients without IHA were hypoglycemic for a significantly shorter time (1.93±2.23 hours) at the beginning of the study (p<0.01). Area under the curve (AUC) for hypoglycemia and number of low excursions at the beginning of the study were 1.81±0.95 and 8.33±3.60, respectively for the IHA group and significantly less (p<0.01) for the others with values of 0.23±0.31 and 2.68±2.05, respectively.

In the patients with IHA the proprotion of time spent with a blood glucose of <70 mg/dL for the postprandial periods were; 19.1% at breakfast, 27.6% at lunch, 24.4% at dinner, 25.4% between 20.00-24.00 hours and 34.6% between 24:00-07:00 hours.

After three months of structured education the IHA patients were hypoglycemic for 4.44±3.78 hours, AUC for hypoglycemia was 0.43±0.47 and the number of low excursions were 5.22±3.99. Though AUC and hypoglycemia duration statistically decreased compared to the initial findings (p=0.01 and p<0.01 respectively), the number of hypoglycemic excursions did not change with structured education. HbA1c levels in IHA patients increased from 7.93±0.90% to 8.20±0.85% with three month educational intervention although this was not statistically significant (p=0.35).

**Figure 1:**
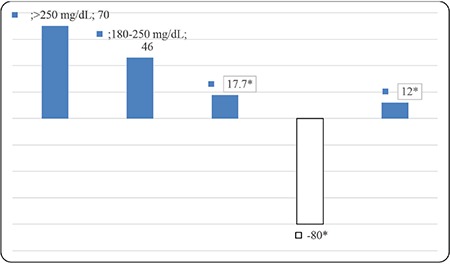
Percentage change in area under the curve (mg/dL x day) after education for impaired hypoglycemia awareness *p<0.05

**Figure 2:**
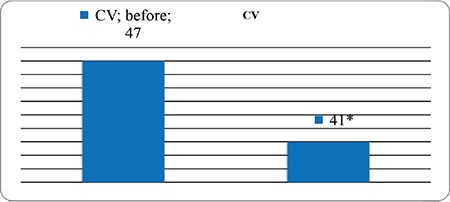
Change in coefficient of variation after education for impaired hypoglycemia awareness *p=0.03, CV: coefficient of variation

When key metrics for CGM were assessed; AUC per 24 hours (mg/dL x day) and time spent for level 1 and level 2 hypoglycemia and percentage of time spent in level 1 hypoglycemia decreased significantly with structured education. AUC per 24 hours (mg/dL x day) and percentage of time spent in level 1 and 2 hyperglycemia did not change (see Table 2 and 3). Percentage of change in AUC (mg/dL x day) for level 1-2 hypo and hyperglycemia and time in range is shown in Figure 1. Level 2 hypoglycemia decreased by 80% while level 1 hypoglycemia increased by 12% and time in range increased by 17.7% (p<0.05, for all of them). Coefficient of variation (CV), which is a measure of primary GV, decreased significantly, although it was unstable at the end of three months, with education in IHA patients (p=0.03) (Figure 2).

## Discussion

Impaired hypoglycaemia awareness is defined as poor alertness and therefore poor responsiveness to the signs and symptoms of hypoglycaemia (3). IHA is a major risk factor for serious hypoglycemia. A significant decrease in autonomic signs has been reported in even very brief periods of hypoglycemia in subjects with hypoglycemia unawareness (8).

IHA is reported frequently in adults with type 1 diabetes (11). In The Diabetes Control and Complications Trial study, 36% of serious hypoglycemia incidents were attributed to hypoglycemia unawareness (12). Cryer et al (13) and Pramming et al (14) reported loss of autonomic signs in 50% of type 1 diabetic adult patients with 15-20 years of disease duration in the questionnaire-based studies they conducted. Gold et al (15) detected IHA in 29 cases (48%) with a mean age of 48.4±11.0 years and a mean duration of 21±8 years. Hepburn et al (11) reported lower rates of IHA in 111 subjects out of 305 (36.4%) type 1 diabetic patients in a questionnaire-based study.

However it is not clear whether frequency of IHA is the same among pre-pubertal children and adolescents. Gravelling et al (8) carried out a questionnaire study of 98 pediatric diabetic patients assessed by scale. They found hypoglycemia unawareness in 22 cases (22.4%) in subjects with a median age of 8.2 (5.7-10.5) years and mean diabetes duration of 3.2±2.0 years. In a large study of 650 children with type 1 diabetes mellitus which included a questionnaire, IHA was reported in 30% of subjects which is similar to results reported for adults with type 1 diabetes (16). In our study, IHA was detected in 24.3% of 37 children and adolescents with type 1 diabetes mellitus.

Davis et al (17) showed that sex is a risk factor because females are more likely to have a suppressed hormone response to hypoglycemia. It has been suggested that estrogen is an intermediary for this. In our study, six of the nine IHA patients were female and four of the six female patients were at Tanner stage 5. Although the number of IHA patients is too few to draw a conclusion about estrogen, the number of female IHA patients was twice that of males with IHA.

Existence of a relationship between high rates of serious hypoglycemia, a decreased ability to detect hypoglycemia together with prolonged duration of type 1 diabetes and development of IHA has been reported frequently in the adult literature (13,14,15,18,19,20,21,22,23). In our study, IHA was detected in patients with shorter disease duration (27.3%) compared to patients with longer disease duration (20%). This finding may be due to the relatively closer duration of diabetes in the two groups and shorter duration of diabetes as compared to the adult studies.

The adoption of more flexible HbA1c targets, especially for diabetic patients who have a history of serious nocturnal hypoglycemia and those who are unable to express hypoglycemic symptoms at younger ages is needed in order to decrease the frequency of hypoglycemia (6,24). The target value for HbA1c in the ISPAD guidelines is <7%, regardless of patient age (25). However, HbA1c levels are not an indicator for frequency of hypoglycemia. In our study, mean HbA1c and mean glucose levels were lower in the IHA group. Considering lower HbA1c values, mean blood glucose levels and continuous subcutaneous glucose monitoring data, presence of IHA has an association with reduced mean blood glucose levels and decreased HbA1c levels. Although not statistically significant, hypoglycemia unawareness tends to occur more frequently in the group with lower HbA1c levels.

In the Type 1 Diabetes Exchange study, the frequency of serious hypoglycemia was lower in pump users (26). It was thought that insulin pump therapy decreased HbA1c without increasing hypoglycemia frequency and the risk of hypoglycemia unawareness (26). In our study only two of the nine IHA patients were on pump therapy without sensors.

Gold et al (15) reported that participants usually experienced hypoglycemic symptoms in the morning. These patients stated awareness of neuroglycopenic symptoms during hypoglycemia. In our study, when subjects were asked the question “Do you experience hypoglycemia signs?”, among subjects with hypoglycemia unawareness diagnosed with continuous subcutaneous glucose monitoring, 21.6% replied ‘yes, I do experience’ and 2.7% replied ‘I sometimes experience’. Not one of the subjects said that they were unaware of hypoglycemia. According to continuous subcutaneous glucose monitoring data over 24-hours, it was evident that subjects who had IHA, mostly experienced hypoglycemia between 24:00-07:00 hours (34.6%) with a further 27.6% detected in the postprandial period at noon and this dropped further to 25.4% between 20:00-24:00 hours. Among subjects who did not experience IHA with continuous subcutaneous glucose monitoring, 54.1% said “I do experience” where 16.2% said “I do not experience”. The difference between continuous subcutaneous glucose monitoring data and answers to the question “Do you experience hypoglycemia signs?” suggested that symptoms indicating hypoglycemia were not noticed, individuals’ perceptions of indications were insufficient for detection of hypoglycemia and individuals replied to the questionnaire based on their emotions at the time of survey rather than their true experience. Continuous subcutaneous glucose monitoring data is more robust because of the elimination of subjective impressions and being reliably quantitative.

Avoiding hypoglycemia for three weeks is sufficient for the abolition of IHA and partial restoration of the adrenal response to hypoglycemia (6,22,27,28). In our study, hypoglycaemia awareness improved at the end of a three month structured training programme which included hypoglycemia and insulin management, safe exercise management and increased target blood glucose levels.

In the Hypo COMPaSS Trial GV was improved within 24 weeks in adults with long-standing type 1 diabetes, complicated by IHA and recurrent severe hypoglycemia, with the help of education based intervention shown by blinded CGM (29). In the study IN CONTROL real time CGM increased time spent in normoglycaemia and reduced severe hypoglycaemia in adult patients with type 1 diabetes and impaired awareness of hypoglycaemia, compared with self monitoring blood glucose (30). In our study we have shown that in type 1 diabetes mellitus with structured education, frequency of level 2 hypoglycemia decreased with more time spent in normoglycemia and produced less glucose variability, as shown by decreased CV, without a change in metabolic control assessed by HbA1c.

### Study Limitation

Shortness of the follow-up period and the low number of cases can be listed as the limitations of this study.

## Conclusion

We have shown that professional CGM is a valuable tool to diagnose impaired awareness of hypoglycemia and that GV can be improved in pediatric type 1 diabetes patients complicated by IHA with the help of education-based intervention.

## Figures and Tables

**Table 1:**
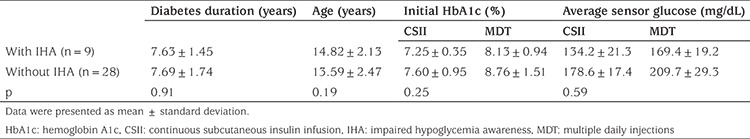
Hemoglobin A1c, diabetes duration, age and mean blood glucose levels of patients with and without impaired hypoglycemia awareness

**Table 2:**
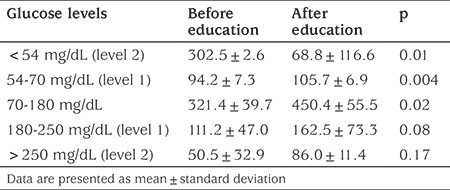
Area under the curve per 24 hours (mg/dL x day)

**Table 3:**
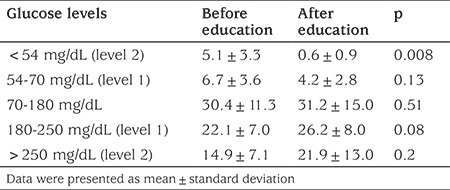
Percentage of time spent with glucose levels in specific glucose ranges
